# The Effect of *Camellia sinensis* on Wound Healing Potential in an Animal Model

**DOI:** 10.1155/2013/386734

**Published:** 2013-06-20

**Authors:** Fatemeh Hajiaghaalipour, M. S. Kanthimathi, Mahmood Ameen Abdulla, Junedah Sanusi

**Affiliations:** ^1^Department of Molecular Medicine, University of Malaya Centre for Proteomics Research (UMCPR), Faculty of Medicine, University of Malaya, 50603 Kuala Lumpur, Malaysia; ^2^Department of Biomedical Science, Faculty of Medicine, University of Malaya, 50603 Kuala Lumpur, Malaysia; ^3^Department of Anatomy, Neuroscience Research Group, Faculty of Medicine, University of Malaya, 50603 Kuala Lumpur, Malaysia

## Abstract

*Camellia sinensis* (tea) is reported to have health benefits, including the building of healthy skin. This study evaluated the effects of topical application of *Camellia sinensis* extract on the rate of wound closure and the histology of wound area. A uniform area of 2.00 cm in diameter was excised from the neck of adult male Sprague Dawley rats. The animals were topically treated with 0.2 mL of vehicle (CMC), Intrasite gel (positive control), or 200 and 400 mg/mL of extract. Wounds dressed with the extract and Intrasite gel healed significantly earlier than those with vehicle. Histological analysis of the wound area after 10 days showed that wounds dressed with the extract had less scar width when compared to the control. The tissue contained less inflammatory cells and more collagen and angiogenesis, compared to wounds dressed with vehicle. In this study, *Camellia sinensis* showed high potential in wound healing activity.

## 1. Introduction

A source of active and efficient medicinal agents has been provided in nature. Based on the usage of natural products in traditional medicine, a large number of modern medicines have been extracted from natural sources [1].

The use of herbal therapies for caring of wounds and injuries has been popular since ancient civilisations. In contrast to only 1–3% of modern drugs being used for the treatment of wounds and skin disorders, almost one-third of all traditional medicines in use are for this treatment [2].

Wounds are physical injuries that result in breakage of the skin. Wound healing is a natural response to tissue injury, consisting of a multifactorial and complex cascade of events involving various cellular, molecular, and biochemical processes and resulting in the healing of the wound and the restoration of the intact functional barrier [3, 4]. Inflammation, cell proliferation, angiogenesis, epithelialisation, wound contraction, and matrix remodeling is the sequence of several basic processes. This multifactorial sequence of processes starts from the moment of injury and continues for varying periods of time, the time period depending on the extent of the injured area and the health status of the injured individual [5].

The wound healing process is generally categorised into three integrated and overlapping phases: the inflammatory phase, which consists of the establishment of homeostasis and inflammation, the proliferative phase, which consists of granulation, contraction, and epithelialisation, and the remodelling phase or resolution, which eventually determines the strength and appearance of the healed tissue [2]. Studies have shown that several natural products, containing active compounds like triterpenes, alkaloids, flavonoids, and other biomolecules, promote the process of wound healing by influencing one or more phases of the healing process [4, 6]. Another study reported that medicinal plants affect the many phases of the healing process, such as coagulation, inflammation, fibroplasia, collagenation, epithelialization, and wound contraction [7].

Second only to water, tea is one of the most consumed beverages in the world. Tea has been reported to contain a number of chemical constituents with medicinal and pharmacological properties [8]. Tea contains relatively high levels of flavonoids, including catechins and other polyphenols. This antioxidant and free radical scavenging activity has been suggested to be the cause of the various health benefits associated with tea drinking. The tea plant, *Camellia sinensis*, is a member of the Theaceae family, and white, green, oolong, and black teas are produced from its leaves.

White tea is an unfermented tea made from the buds and young tea leaves, which are harvested once a year in the early spring. Unlike black and oolong teas, white and green tea production does not oxidise the young tea leaves [9, 10]. White and green teas are produced by steaming fresh leaves at high temperatures, leaving the polyphenol content intact, but inactivating enzymes that would oxidise the teas [11]. Oolong tea undergoes partial fermentation before drying and has a taste and color somewhere between green and black teas. Black tea, however, undergoes fermentation, while green tea does not [12, 13]. Numerous *in vitro* and *in vivo* studies have reported beneficial health properties of tea and their phenolic compounds [14, 15]. However, there has not been much attention given to the properties of white tea. In our ongoing studies white tea has demonstrated significant antioxidant and anticarcinogenic properties. Therefore, the present study was undertaken to evaluate the effectiveness of topical application of the methanolic extract of Silver Needle white tea on the rate of wound closure and the histology of the wound area.

## 2. Materials and Methods

### 2.1. Dressing

Intrasite gel (purchased from the University of Malaya Medical Centre Pharmacy) was used as a positive control. Intrasite gel (trademark, Smith and Nephew Healthcare Ltd) is a colorless transparent aqueous gel, containing 2.3% of a modified carboxymethyl cellulose polymer together with propylene glycol (20%). It functions as a humectant and preservative. When placed in contact with a wound, the dressing absorbs excess exudates and produces a moist environment at the surface of the wound, without causing tissue maceration. Intrasite gel, an amorphous hydrogel, gently rehydrates necrotic tissue and facilitates autolytic debridement, while loosening and absorbing slough and exudates, and thus clearing the way for effective wound healing. It is designed for wounds that are granulating and epithelialising. It is also used to provide the optimum moist wound management environment during the later stages of wound closure. It is nonadherent and does not harm viable tissue or the skin surrounding the wound. This makes the use of Intrasite gel ideal for every stage in the wound management process [16]. 

Lignocaine HCL (2%, 100 mg/5 mL), a local anaesthetic, was purchased from the Experimental Animal House, Faculty of Medicine, University of Malaya (Delta Veterinary Laboratory PTY LTD, NSW). A 1 mL of lignocaine was injected subcutaneously.

### 2.2. Tea Extract Preparation

Silver Needle white tea was purchased from the local market. Tea infusions were prepared by placing 2 g of tea leaves in 100 mL methanol at room temperature for 2 hours and filtered through Whatman filter paper no. 1. The resulting solvent extract was concentrated using a rotary evaporator. The concentrated extracts were stored at −20°C until further analysis. 

### 2.3. Experimental Animals

Twenty-four healthy adult male Sprague Dawley rats, 8 weeks old and weighing 200 to 250 g, were obtained from the Experimental Animal House, Faculty of Medicine, University of Malaya. This study was carried out according to the criteria outlined in the “Guide for the Care and Use of Laboratory Animals” published by the National Institutes of Health. The rats were divided randomly into 4 groups of 6 rats each. Each rat was housed separately (one rat per cage), and the animals were maintained on standard pellet diet and tap water. The study was approved by the Ethics Committee for animal experimentation, Faculty of Medicine, University of Malaya, Ethics no. PM/22/11/2011/FH (R).

### 2.4. Experimentally Induced Excision Wounds

The rats were inflicted with excision wounds as described by Mughrabi et al. [17]. Briefly, the animals were anaesthetised with 0.09 mL of ketamine by i.m. injection (30 mg/kg, 100 mg/mL) and 0.01 mL of Xylazil by i.m. injection (3 mg/kg, 100 mg/mL) prior to creation of the wounds. The skin was shaved using an electrical clipper, disinfected with 70% alcohol, and injected with 1 mL of lignocaine HCl s.c. injection (2%, 100 mg/5 mL). An area of uniform wound 2.00 cm in diameter was excised using a circular stamp and sterile scissors, from the depilated, ethanol-sterilised nape of the dorsal neck of the 24 rats. Incision of the muscle layer was avoided during the procedure so that the tension of the skin would not be affected. The wound area was measured immediately by placing transparent paper over the wound and tracing out the area. This was subsequently placed on a 1 mm^2^ graph sheet. The area of the counted squares was calculated in square millimeters and the area was recorded as described by Zahra et al. [18].

### 2.5. Topical Wound Application

Wounds of Groups I and II animals were topically treated with 0.2 mL of 200 (high dose) and 100 (low dose) mg/mL of a methanolic extract of white tea in vehicle, respectively. Wounds of Groups III animals were topically treated with 0.2 mL Intrasite gel twice daily as a reference standard control. Wounds of Group IV rats were topically treated with 0.2 mL of vehicle, carboxymethyl cellulose (CMC) in normal saline (2%), twice daily as control group [19].

### 2.6. Estimation of Wound Healing (Wound Closure)

The wound closure area of each animal was assessed by tracing the outline of the wound on days 5 and 10 after wounding surgery and the wound closure rate was expressed as the percentage of wound area compared with that on postoperative day as described previously [18]. The wound closure rate was expressed as the percentage of wound area compared with that on post-operative day; that is, the change in wound size was expressed as a percentage of the original wound size:

(1) contraction =Initial  wound  size−Specific  day  wound  sizeInitial  wound  size×100.

### 2.7. Histological Evaluation of Healed Wounds

Skin tissue samples of all groups were immediately fixed after animal dissection, in 10% buffered formalin, dehydrated, and processed using a paraffin tissue processing machine for paraffin sectioning. The wound tissues were cut 5 *μ*m thick perpendicular to the wound, stained with standard haematoxylin and eosin (H&E) and Masson's trichrome (MT), and examined using an Olympus BX60 light microscope and captured using an Olympus XC10 camera (Tokyo, Japan). The evaluated parameters were epithelialisation, inflammatory cell infiltration, fibroblast proliferation, neovascularisation, and collagen deposition, which were assessed individually by using a numerical scale. Stained sections were examined under a light microscope and were graded in a blind fashion on 3 slides per animal, using the modified 0 to 4 numerical scale as described by other researchers [20, 21]. The scores were 0 for absence, 1 for occasional presence, 2 for light scattering, 3 for abundance, and 4 for confluence of cells or fibers.

### 2.8. Statistical Analysis

Experimental results are presented as means ± SD of six animals in each group. Statistical analysis was performed by using SPSS (18.00 version) statistical software. The effect of extracts on the wound was analysed by one-way ANOVA. *P* values < 0.05 were considered significant. 

## 3. Results 

**Figure 1:**
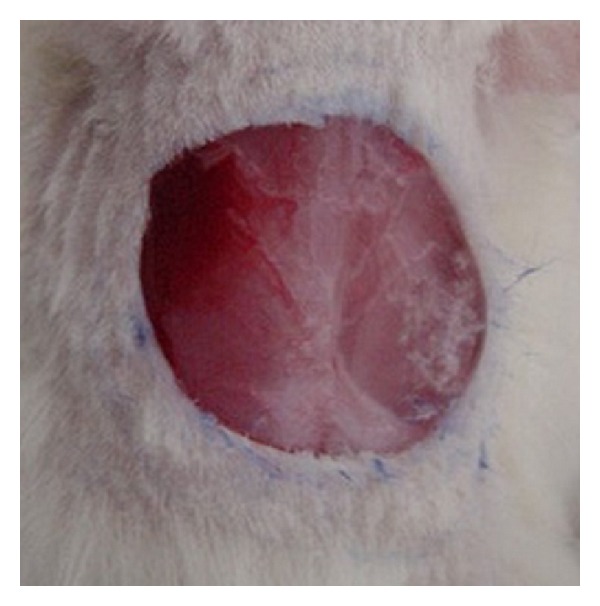
Excision skin wound (2.00 cm) on surgery day before treatment.

**Figure 2:**
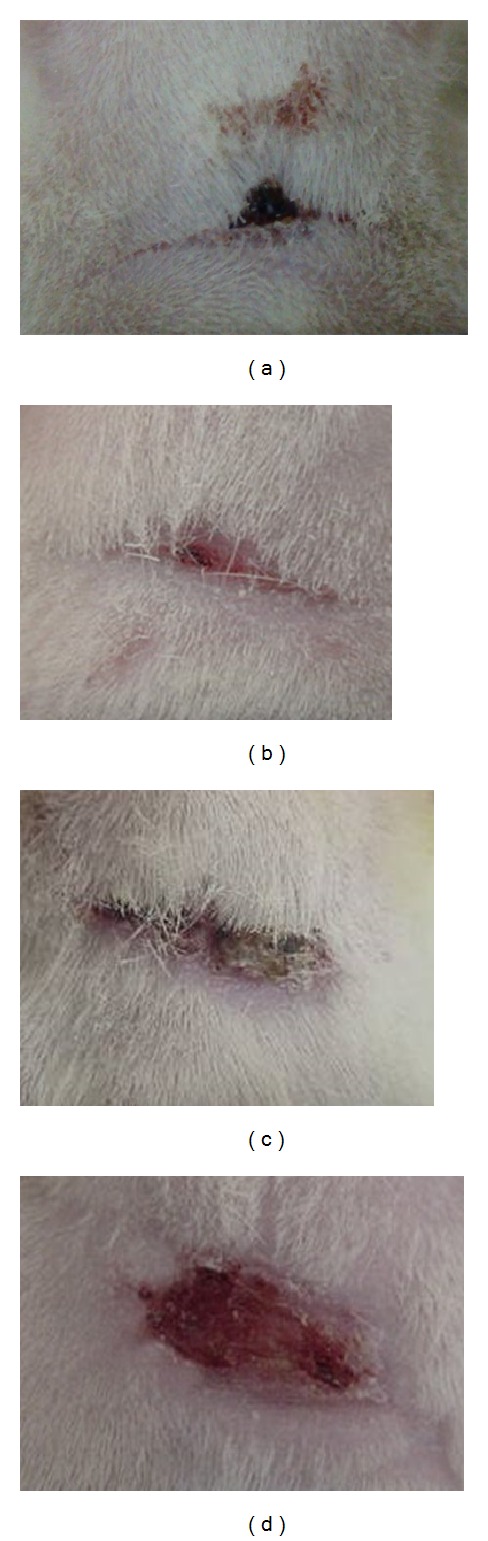
Macroscopic appearance of wound on day 10 after surgery. (a) A 200 mg/mL of white tea treated group, (b) 100 mg/mL of white tea treated group, (c) Intrasite gel treated group, and (d) control group (wound dressed with placebo, CMC).

Figure 1 shows the macroscopic appearance of the excised skin wound, after surgery. The macroscopic examination on day 10 after surgery is presented in Figure 2. As shown in the figure, the 3 groups (high-dose treated, low-dose treated, and Intrasite gel-treated, that is, Figures 2(a), 2(b), and 2(c), resp.) showed a narrow scar at wound closure compared with the control group, that is, wound dressed with placebo (CMC), Figure 2(d), which showed incomplete wound healing.

 The change in the wound size was expressed as a percentage of the wound area on days 5 and 10 after surgery compared with the wound area on day 0 (100%) in the different groups (Table 1). Wounds dressed with white tea extract or with reference standard control showed considerable signs of dermal healing and significantly healed faster compared to the group that received the placebo control treatment (CMC in normal saline). Throughout the experiment, the percentage of wound healing in the control (CMC) group was significantly lower than that in the treated groups or that in the group treated with Intrasite gel (standard) on day 5 after surgery (Table 1). The percentage of healing in the high dose group was significantly higher than those of the low-dose-treated group, the reference standard group, and the control group on day 10 after surgery, although the percentage of healing in the placebo (CMC) control group was significantly lower than those of the extract-treated groups and the reference standard control (*P* < 0.05). There was no significant difference between low-dose-treated groups and standard control group on day 10 after surgery (*P* > 0.05). The effect of low dose was similar to that of the standard.

**Figure 3:**
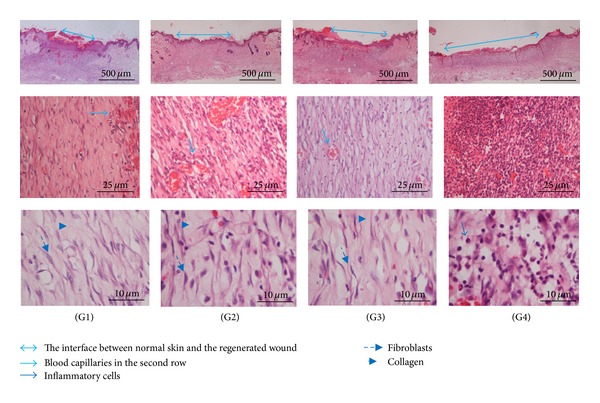
Histology of wound area stained with hematoxylin and eosin on day 10 after surgery. Photomicrographs of wound tissues in different magnification are shown in the rows.

**Figure 4:**
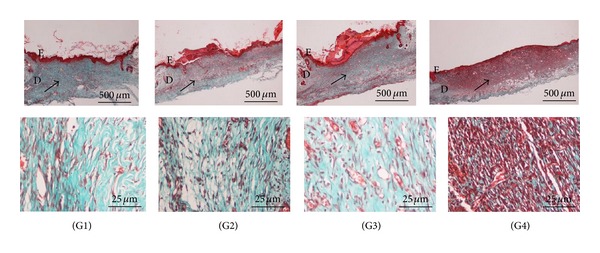
Histological sections of healed wound on day 10 after-surgery stained with Masson's trichrome. Different magnifications of the wound are shown in the rows. E: epidermis, D: dermis. The arrows in the upper row show the different levels of collagen deposition, represented by the intensity of the green color.

The histology of wound area on day 10 after surgery showed that wounds dressed with the extract showed comparatively less scar width at wound closure compared to the control group (Figures 3 and 4). In all the groups, with respect to the control group, inflammatory cell infiltration was less intense and the difference between the groups was statistically significant (*P* < 0.05). In Groups I, II, and III, there was no significant difference in inflammatory cell infiltration (*P* > 0.05). In extract-treated groups and the standard treated group, comparatively, more collagen and fibroblasts were seen (Figure 3). Proliferation of blood capillaries and angiogenesis were significantly more pronounced in Groups I, II and III when compared with group IV.  Figure 4 shows Masson's trichome staining of the wound after 10 days. In both groups II (low-dose treated) and III (Intrasite gel treated), the presence of collagen and fibroblasts is similar to Group I (high-dose treated), although the photomicrograph of Group I shows a higher density of collagen and more fibroblast cells, though this was not significant (Table 2). The CMC-treated group (Group IV) has significantly (*P* < 0.05) less collagen, less fibroblasts, and less blood capillaries in comparison with the other groups.

## 4. Discussion

In this study an excision wound model was used for the assessment of the wound healing activity of *Camellia sinensis*, specifically white tea. Wound healing progression is comprised of a systematic process of events starting from the moment of injury, that is, the inflammatory phase (the establishment of homeostasis and inflammation), the proliferation phase (granulation, contraction, and epithelialisation), and finally, the remodeling phase, which determines the strength and appearance of the healed tissue [3, 22].

The outcome of this study demonstrates that the wound contracting ability of the white tea extract on an excision wound model was significantly greater than that of the vehicle itself. 

Histopathological studies of the wound healing process are normally used for evaluating the efficacy of pharmacological products which promote and accelerate dermal skin substitutes. The histological results of this study show that the granulation tissue in tea extract-treated groups contained comparatively less inflammation and more collagen and angiogenesis. The histopathological examination of the wound sections in the control group exhibited a wide area of ulceration (presumably containing fibrinous exudates) and inflammatory cells and a mild degree of vascularisation with congestion in the dermis, which indicate that healing was not complete. It can be concluded that topical application of the tea extract at a high concentration (200 mg/mL) significantly increased the fibroblast growth, collagen synthesis, and thus the healing process by accelerating the rate of wound healing.

In the present study haematoxylin and eosin (H&E) staining was used (Figure 3) as it is the most common method in histopathological studies. Histological sections stained with haematoxylin and eosin (H&E) on day 10 after-surgery showed that wounds dressed with the extract showed comparatively less scar width at wound closure compared to the control group (Figure 3). The histology of wound area in Intrasite gel- and low-dose extract-treated groups showed similar results. In both groups, the extent of epithelialisation, neovascularisation, and fibroblast proliferation and the presence of collagen are similar. Histological analysis showed that the intensities of the collagen (green colour) of Groups I, II, and III are different but not significant (*P* > 0.05). Proliferation of blood capillaries and angiogenesis were significantly higher in Groups I, II, and III when compared with Group IV (*P* < 0.05).

Although H&E staining is the most popular staining method, the stain is not able to differentiate important histopathological changes in the wound healing process, such as collagen deposition. An alternative staining method, modified Masson's trichrome staining (MT), was used to differentiate the important morphological parameters for wound healing assessment. The results of the MT staining in this study confirm that the presence of collagen in Intrasite gel- and white tea extract-treated groups is significantly higher when compared to the control group. As shown in Figure 4 for the results of MT staining (presented at two different magnifications, 2x and 40x), the intensity of the green color represents collagen density. Fibroblasts produce collagen in skin which plays an important role in preserving the anatomic integrity of wound healing. MT staining showed differences but this was not significant for fibroblast proliferation, collagen deposition, and neovascularisation in Intrasite gel- and white tea extract-treated groups (*P* > 0.05). However, the difference in collagen deposition between the treated groups mentioned above and in the CMC-treated group is significant. The inflammatory cells in Groups I–III were significantly less (*P* < 0.05) than those in Group IV.

The results showed a trend of progressive construction of tissues surrounding the wound from day 5 onwards (Table 1) especially for the Intrasite gel- and tea extract-treated groups. This was significantly different (*P* < 0.05) from the control group. There was no significant difference in the semiquantitative histopathological evaluation of wound healing between the groups treated with Intrasite gel and extract treated groups as shown in Table 2. 

Inflammation is the first response during the healing period as a defence mechanism of the tissue, although a long duration in the inflammatory phase can cause a delay in the healing process [23]. The results of this study suggest that application of *Camellia sinensis* extract shortens the inflammation period, in order to shorten the healing time.

Transforming growth factor-beta1 (TGF-β1) has been shown to be expressed during the wound healing process. TGF-β1 acts by the stimulation of fibroblast proliferation and differentiation, collagen production, wound contraction, and connective tissue growth factor gene expression [24]. *α*3β1 integrin has also been shown to be highly expressed during reepithelialization [25]. Epigallocatechin-3-gallate (EGCG), a major component of tea, has been shown to enhance the role and expression of TGF-β1 [26]. EGCG has also been reported to enhance the expression of vascular endothelial cell growth factor and angiopoietin-1 protein expression [27].

A previous study has shown that healing can be accelerated and enhanced by the use of specific wound dressing or care product and techniques, and that it is not a passive process [28]. It has been observed that plant constituents can significantly accelerate the healing process and improve the quality of wound healing [29]. Numerous studies have shown that plant compounds could potentially be therapeutic agents to treat wounds [30, 31]. The findings of this study are also in line with previous studies reported by various authors [18, 19, 31]. 

It is generally accepted that reactive oxygen species (ROS), because of their harmful effects on cells and tissues, are noxious for the wound healing process [32]. Antioxidants/free radical scavengers play a significant role in the process of wound healing by improving the healing time and the appearance of the healed tissue, while protecting tissues from oxidative damage. The wound healing action of *Camellia sinensis* may be due to the phytoconstituents present in the plant (high flavonoid and phenolic content) and also high free radical scavenging activity.

The chemical constituents of tea leaves include polyphenols (catechins and flavonoids), alkaloids (caffeine, theobromine, theophylline, etc.), volatile oils, polysaccharides, amino acids, lipids, vitamins (e.g., vitamin C), and inorganic elements (e.g., aluminium, fluorine, and manganese). The polyphenols are the group of compounds that are primarily responsible for the beneficial healthful properties of tea. The flavonoids have antioxidant, anti-inflammatory, antiallergic, and antimicrobial effects. All of these constituents play important roles in the maintenance of human health and also in wound healing [14].

The oral bioavailability of catechins from tea has been reported to be about 5 to 50 times less than that in *in vitro *studies [33] although an increase in human plasma antioxidant activity has been shown after the consumption of tea [34]. Catechins were present in plasma at micromolar levels after oral consumption of tea. Catechins are metabolized and circulate as sulfated, methylated, or glucuronidated derivatives; epigallocatechin gallate and epicatechin gallate were mostly present in plasma as the free form. 

As our results show that treated groups healed faster, showed less scarring, more blood capillaries, less inflammatory cells, and more fibroblasts and collagen, we propose that white tea might enhance the wound healing process by increasing the rate of various phases, such as cell proliferation, angiogenesis, and collagen formation. 

## 5. Conclusion

This study showed that the wound area of wounds dressed with white tea extract had relatively less scar width at wound closure. Granulation tissue contained less inflammatory cells and more collagen with angiogenesis compared to wounds dressed with vehicle. *Camellia sinensis* shows high potential in wound healing activity, especially at higher concentrations.

## Figures and Tables

**Table 1:** Effect of methanolic extract of *Camellia sinensis* on percentage (%) of wound healing in Sprague Dawley rats.

Animal groups	Treatment	Percentage of wound healing (mean ± SD) on days after surgery	
		Day 5	Day 10
Group 1	High dose, 200 mg/mL	61.9 ± 6.70^a^	97.7 ± 0.94^a^
Group 2	Low dose, 100 mg/mL	62.3 ± 3.99^a^	94.5 ± 1.12^b^
Group 3	Intrasite gel	60.3 ± 6.70^a^	95.4 ± 1.29^b ^
Group 4	CMC in normal saline	45.0 ± 1.98^b ^	86.5 ± 2.40^c ^

Mean values (*n* = 6) followed by different letters (a, b, and c) in a column are significantly different (*P* < 0.05).

**Table 2:** The median histopathologic scores of wound healing were determined in the extract-treated and control groups by using a modified 0 to 4 numerical scale. The scores were 0 for absence, 1 for occasional presence, 2 for light scattering, 3 for abundance, and 4 for confluence of cells or fibres.

Groups	Epithelialisation	Inflammatory cell infiltration	Fibroblast proliferation	Neovascularisation	Collagen deposition
G1 (high dose)	3.83 ± 0.41^a^	1.50 ± 0.55^b^	3.50 ± 0.55^a^	2.67 ± 0.52^a^	3.83 ± 0.41^a^
G2 (low dose)	3.17 ± 0.41^a^	1.83 ± 0.75^b^	3.17 ± 0.75^a^	2.00 ± 0.63^a^	3.33 ± 0.52^a^
G3 (Intrasite gel)	3.50 ± 0.55^a^	1.67 ± 0.52^b^	3.33 ± 0.82^a^	2.17 ± 0.75^a^	3.67 ± 0.52^a^
G4 (CMC, control)	0.83 ± 0.41^b^	3.67 ± 0.52^a^	1.00 ± 0.00^b^	0.83 ± 0.75^b^	1.17 ± 0.41^b^

Data are expressed as mean ± standard error (*n* = 6). Different letters (a and b) in a column indicate significant difference (*P* < 0.05).
